# Dual‐Wavelength Laser Therapy for Painful Diabetic Peripheral Neuropathy: A Randomized, Double‐Blind, and Sham‐Controlled Trial

**DOI:** 10.1155/prm/8710857

**Published:** 2026-10-11

**Authors:** Sandra Franco, Cesar Lara, Kirk Gair, Kerry Zang, Jaime Franco, Steve Shanks, Travis Sammons

**Affiliations:** ^1^ Franco & Co–Podiatric Medicine & Surgery Miramar Florida USA; ^2^ Weight Loss & Wellness Dunedin Florida USA; ^3^ Laser Chiropractic West Covina California USA; ^4^ Regenerative Foot & Ankle Medicine Scottsdale Arizona USA; ^5^ Erchonia Corporation Fountain Inn South Carolina USA

**Keywords:** diabetic peripheral neuropathy, home-use device, low-level laser therapy, neuropathic pain, photobiomodulation, randomized controlled trial

## Abstract

**Background and Aims:**

Painful diabetic peripheral neuropathy (DPN) is common and often inadequately controlled with pharmacological therapy. This study evaluated a clinician‐directed, prescription home‐use dual‐wavelength low‐level laser therapy device for temporary relief of painful DPN.

**Methods:**

In this prospective, randomized, double‐blind, sham‐controlled trial conducted at four independent U.S. clinical sites, 64 adults with painful DPN were randomized 1:1 to active treatment (*n* = 33) or sham treatment (*n* = 31). The active device emitted simultaneous 640 red and 405 nm violet coherent laser light. Participants self‐administered treatment twice daily for 3 weeks, with each session consisting of 5 min per foot. The primary endpoint was the proportion of participants achieving at least a 30% reduction in visual analog scale (VAS) foot pain score from baseline to Week 3. Secondary outcomes included VAS change, neuropathic pain symptoms, treatment satisfaction, durability through Week 7, adherence, blinding integrity, and safety. Participants were required to maintain a stable analgesic and neuropathic‐pain regimen throughout, so the intervention was delivered in addition to existing standard care. The primary endpoint was analyzed in the intent‐to‐treat population, with single imputation for the four participants who withdrew before receiving a device.

**Results:**

At Week 3, 72.7% of active‐treated participants met the primary responder criterion compared with 32.3% of sham‐treated participants, a between‐group difference of 40.5 percentage points (95% CI 18.1 to 62.9) (*p* = 0.0024). Mean VAS reduction was greater with active treatment than sham treatment (−33.4 vs −20.0 points; *p* = 0.026). Improvements were sustained through Week 7, with favorable symptom trends, higher satisfaction, and no serious device‐related adverse events. Improvement on the neuropathic pain symptom inventory was greatest for paresthesia/dysesthesia, paroxysmal pain, and burning pain and was not evident for evoked pain.

**Interpretation:**

Prescription home‐use dual‐wavelength laser therapy was well tolerated and produced clinically meaningful short‐term reductions in foot pain in adults with painful DPN at four independent clinical sites. Because assessment ended four weeks after treatment completion, the findings support short‐term pain relief, and the intervention is best positioned as a complementary, nonpharmacological modality used alongside standard care.

**Trial Registration:** ClinicalTrials.gov identifier: NCT05291975

## 1. Introduction

Diabetic peripheral neuropathy (DPN), most commonly presenting as distal symmetric polyneuropathy, is among the most common complications of diabetes and may affect up to half of patients during the course of disease [1, 2]. Painful DPN impairs sleep, mobility, daily function, and quality of life, and it contributes substantially to healthcare utilization and lost productivity [3–5]. Despite available pharmacological options, many patients experience incomplete relief or adverse effects that limit long‐term use.

Current medications used for painful DPN include gabapentinoids, serotonin‐norepinephrine reuptake inhibitors, tricyclic antidepressants, and opioids. These therapies may be associated with dizziness, sedation, cognitive impairment, gastrointestinal adverse effects, and, for opioids, dependence and tolerance [6–8]. The need remains for safe, nonpharmacological adjunctive therapies that can be integrated into routine care and used consistently by patients with chronic symptoms.

Chronic disease management increasingly occurs outside traditional clinical settings. The U.S. Food and Drug Administration describes home‐use medical devices as devices intended for use outside professional healthcare facilities by patients, caregivers, or family members, with appropriate involvement of qualified healthcare professionals when needed [9]. For painful DPN, a prescription home‐use intervention may reduce the need for frequent office visits and provide a practical approach for patients with mobility limitations while preserving clinician oversight.

Photobiomodulation, also referred to as low‐level laser therapy (LLLT), delivers nonthermal light energy that can influence mitochondrial activity, oxidative stress signaling, vascular signaling, inflammation, and nociceptive pathways [10–12]. These effects are biologically plausible for painful DPN because diabetes‐associated mitochondrial dysfunction, impaired axonal energy metabolism, and oxidative stress contribute to peripheral nerve injury and pain [3, 13].

The present study evaluated the efficacy and safety of a dual‐wavelength coherent LLLT device emitting simultaneous 640 nm red and 405 nm violet laser light for clinician‐directed at‐home treatment of painful DPN. The trial was designed as a randomized, double‐blind, sham‐controlled study with a responder‐based primary pain endpoint and follow‐up four weeks after treatment completion. The clinical outcomes from this study were subsequently used to support U.S. FDA marketing clearance under 510(k) K251903.

## 2. Materials and Methods

### 2.1. Study Design and Oversight

This was a prospective, randomized, double‐blind, sham‐controlled multicenter clinical trial conducted at four independent U.S. clinical sites. The four participating sites were geographically and clinically distinct outpatient practices located in Miramar, Florida; Dunedin, Florida; West Covina, California; and Scottsdale, Arizona. The first participant was enrolled on June 14, 2022, and the last on September 25, 2024, and the final Week 7 follow‐up assessment was completed on November 14, 2024; the trial ended after the planned enrollment target had been reached rather than in response to any interim finding. The protocol was approved by an independent institutional review board (WCG Copernicus Group; IRB Tracking #20220447), and all participants provided written informed consent before enrollment. The trial was conducted in accordance with the Declaration of Helsinki [14] and applicable U.S. federal regulations governing human‐subject research.

The study was registered on ClinicalTrials.gov before the enrollment of the first participant. This report follows the CONSORT 2025 Statement, which supersedes CONSORT 2010 [15]. A completed CONSORT 2025 checklist, indicating the page on which each item is addressed, is provided as Supporting Information, and participant flow from screening through analysis is presented as a CONSORT flow diagram in Figure 1. There were no changes to eligibility criteria, outcomes, or trial design after the trial commenced, no interim analyses were performed, and no formal stopping guidelines were applied.

**FIGURE 1:**
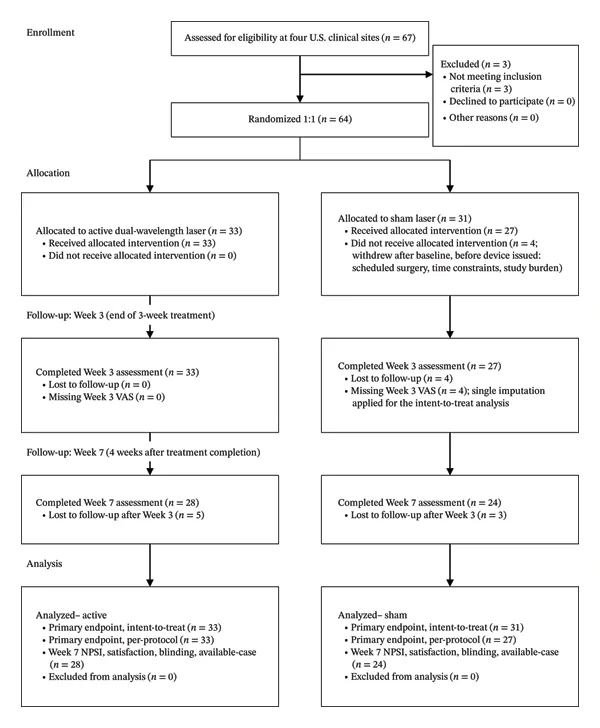
CONSORT 2025 participant flow diagram. Sixty‐four participants were randomized 1:1 at four independent U.S. clinical sites. The intent‐to‐treat population comprised all 64 randomized participants; the per‐protocol population comprised the 60 participants who received the allocated device. Week 7 analyses of NPSI, satisfaction, and blinding were conducted on an available‐case basis (*n* = 52).

### 2.2. Sample Size

The sample size was fixed in advance of enrollment. The protocol anticipated that approximately 55% of participants in the active arm and approximately 20% in the sham arm would meet the individual responder criterion; for that contrast, a two‐sided alpha of 0.05% and 80% power require approximately 29 participants per group in a comparison of two independent proportions, and a target of 64 randomized participants was set to allow for nonevaluable participants. Enrollment stopped when this target was reached. The calculation used the normal approximation for the difference between two independent binomial proportions, computed using the method for comparing two binomial proportions in Rosner, Fundamentals of Biostatistics. To allow for an anticipated 8.3% withdrawal rate, the requirement of 29 evaluable participants per group was inflated by a factor of 1/(1−0.083), giving 32 participants per group and a planned enrollment of 64. The 35‐percentage‐point difference between the two anticipated responder rates was carried forward unchanged as the prespecified success criterion for the primary endpoint, and no interim sample‐size re‐estimation was performed.

### 2.3. Participants

Eligible participants were adults aged 18 years or older with a diagnosis of diabetes‐induced peripheral neuropathy confirmed by a suitably qualified and licensed health professional within the prior 6 months. Participants were required to have constant foot pain for at least 3 months, a VAS foot pain score of at least 50 on a 0–100 mm scale at the screening qualification visit, ability to read and write English, willingness to maintain a stable analgesic regimen with no upward dose titration for at least 14 days before enrollment and throughout study participation, and willingness to refrain from nonstudy pain‐management therapies during the study. Participants were instructed to withhold analgesic use for at least 6 h before scheduled VAS pain assessments.

Sites were eligible if the principal investigator held a current professional license appropriate to the treatment of DPN, provided a curriculum vitae and documentation of Good Clinical Practice training, had the staff and facilities to conduct the protocol, held current institutional review board approval, and completed site initiation visit training before enrolling participants. The four participating sites were U.S. outpatient specialty practices. All treatments were self‐administered by participants at home, so no clinical personnel delivered the intervention; site staff issued the device, provided the written proper use reference guide, and conducted the in‐clinic study assessments.

Exclusion criteria included pregnancy or suspected pregnancy; open wounds, ulcers, sores, or cuts around the feet; cancerous growths around the feet; or difficulty with hand dexterity sufficient to impair self‐administration of the device, including severe arthritis, multiple sclerosis, cerebral palsy, Parkinson’s disease, or Huntington’s disease.

### 2.4. Patient and Public Involvement

Patients and members of the public were not involved in the design, conduct, or reporting of this trial. Participants contributed the primary and secondary outcome data as patient‐reported measures and were asked at the end of treatment and at follow‐up to rate their satisfaction with any change in foot pain but were not involved in setting the research question, selecting the outcome measures, or preparing this report.

### 2.5. Concomitant Medications and Other Pain Therapies

Concomitant therapy was managed identically in the two arms. Participants entered the trial on their existing analgesic and neuropathic‐pain regimens and were required to keep those regimens stable, with no upward dose titration and no introduction of a new agent, for at least 14 days before enrollment and for the full duration of study participation. Participants also agreed to refrain from nonstudy pain‐management therapies during the trial, including physical therapy, acupuncture, chiropractic manipulation directed at the feet, transcutaneous electrical nerve stimulation, injections, and other device‐based treatments. The study intervention was therefore delivered in addition to standard care in both arms and was not compared with, substituted for, or withheld from any participant’s pharmacological therapy. No rescue medication was supplied by the sponsor. Concomitant medications were recorded at screening by drug name, dose, and frequency and were reviewed against the participant’s treatment administration log at each subsequent study visit; any change in an analgesic or neuropathic‐pain medication was documented and classified as a protocol deviation. Because analgesic use can mask the underlying pain state, participants withheld analgesics for at least 6 h before every scheduled VAS assessment. Baseline concomitant medication use by therapeutic class is reported for each arm in Table 1.

**TABLE 1:** Baseline demographic, pain, and concomitant medication characteristics of the study population (intent‐to‐treat population, *n* = 64).

Characteristic	Active (*n* = 33)^∗^	Sham (*n* = 31)^∗^
Age, years, mean ± SD	67.4 ± 8.4	64.7 ± 9.1
Sex, *n* (male/female)	18/15	16/15
Insulin dependent, *n* (yes/no)	11/22	10/21
Duration of diabetes, years, mean ± SD	15.6 ± 12.4	10.2 ± 7.9
Baseline VAS pain score, mm, mean ± SD	76.5 ± 10.5	76.6 ± 14.1
Duration of painful foot symptoms, months, mean ± SD	93.3 ± 89.9	79.5 ± 97.6
None	14 (42%)	12 (39%)
Gabapentin	15 (45%)	14 (45%)
Metformin	3 (9%)	2 (6%)
B‐vitamin complex (supplement)	—	2 (6%)
Dulaglutide	1 (3%)	—
Acetaminophen	1 (3%)	—
Tramadol	1 (3%)	—
Ibuprofen	1 (3%)	—
Duloxetine	1 (3%)	—
Hydrocodone	1 (3%)	1 (3%)
Fluoxetine	—	1 (3%)
Methadone	—	1 (3%)
Tadalafil	—	1 (3%)
Nerve Renew (proprietary supplement)	1 (3%)	—

*Note:* Medications listed are those taken at enrollment for relief of diabetic peripheral neuropathy foot pain, reported by generic name; brand names recorded on the source documents were mapped to their generic equivalents (Neurontin to gabapentin, Vicodin to hydrocodone, Tylenol to acetaminophen, Trulicity to dulaglutide, and Cialis to tadalafil). Gabapentin doses recorded were 100, 300, 400, 600, and 800 mg; doses of other agents are not reported. Concomitant medications taken for indications other than foot pain were recorded separately and are not shown here.

Abbreviations: SD, standard deviation; VAS, visual analogue scale.

^∗^Values are *n* (%) unless otherwise indicated; medication totals exceed the group size because some participants reported more than one medication.

### 2.6. Randomization and Blinding

Participants were randomized 1:1 to active or sham devices using variable block randomization, with block sizes of two, four, and six used at random, and the allocation sequence generated by a computer and concealed from participants. A single employee at the sponsor site held the key to linking the group label to an active or sham device until analysis of the final study dataset was complete. Active and sham devices were visually similar and operated similarly. Blinding validity was evaluated by asking participants at Week 3 and Week 7 to indicate their perceived treatment allocation as a forced binary choice between active and sham, with no ‘uncertain’ option, together with the reason for their determination. Blinding was quantified as the number and proportion of participants in each arm who correctly identified their allocation at each assessment, with the association between actual and perceived allocation tested by chi‐square analysis at a two‐sided alpha of 0.05. Bang’s Blinding Index was calculated post hoc for each arm at each assessment as the proportion of correct guesses minus the proportion of incorrect guesses, with 95% confidence intervals derived from the normal approximation; an index of 0 indicates guessing consistent with chance, +1 complete correct identification, and −1 complete incorrect identification. Bang’s index was used in preference to James’s index because the allocation‐perception item offered a forced binary choice with no ‘uncertain’ option. The free‐text rationales were evaluated qualitatively, as prespecified, according to whether they referred to treatment efficacy or lack thereof or to sensory or visual cues or to overheard conversations among study personnel.

### 2.7. Intervention

Participants self‐administered either active treatment or sham treatment at home twice daily for three consecutive weeks, for 5 min per foot per session and 42 planned treatment sessions in total. Study personnel provided standardized device‐use instructions. The active device (EVRL; Erchonia Corporation, Fountain Inn, SC, USA) delivers line‐generated coherent laser beams at 640 red and 405 nm violet wavelengths simultaneously during each treatment session. Sham devices were visually identical in design and operation but emitted nontherapeutic red and violet light using light‐emitting diodes (LED) to support participant and investigator masking. Treatment was applied to the dorsal surface of each foot, with the device held several inches above the skin and never in contact with it. The two line‐generated beams were projected in parallel along the longitudinal axis of the foot, extending from the anterior ankle at the junction of the leg and the dorsum, distally across the mid‐dorsum and the dorsal interspaces, to the toes; at the treatment distance, the approximately 15.2 cm line spanned this field in a single placement. The irradiated corridor therefore overlays the course of the dorsalis pedis artery and the accompanying deep fibular (peroneal) nerve, the arcuate and dorsal metatarsal arteries, the dorsal venous arch, and the terminal branches of the superficial fibular (peroneal) nerve [16]. Complete parameters for the active device are reported in Table 2.

**TABLE 2:** Treatment parameters for the active study device.

Parameter	Red beam	Violet beam
Wavelength	640 ± 10 nm	405 ± 10 nm
Optical power output	7.5 ± 1 mW	7.5 ± 0.5 mW
Beam delivery	Diode laser energy collected and dispersed through line‐generating optics	Diode laser energy collected and dispersed through line‐generating optics
Beam geometry	Line‐generated beam; line length approximately 15.2 cm (6 in)	Line‐generated beam; line length approximately 15.2 cm (6 in)
Power density (irradiance) at the treatment surface	1.5 mW/cm^2^	1.5 mW/cm^2^
Energy density delivered per minute	0.09 J/cm^2^	0.09 J/cm^2^
Treatment time per foot per session	5 min	5 min
Energy density per foot per session	0.45 J/cm^2^	0.45 J/cm^2^
Energy density per foot per day	0.90 J/cm^2^	0.90 J/cm^2^
Cumulative energy density per foot over the treatment course	18.9 J/cm^2^	18.9 J/cm^2^
Laser classification	Class 2 (IEC 60825‐1)	Class 2 (IEC 60825‐1)

Both diodes emit simultaneously throughout each session, giving a combined optical output of 15 mW, a combined energy density of 0.90 J/cm^2^ per foot per session, 1.80 J/cm^2^ per foot per day, and 37.8 J/cm^2^ per foot across the 42‐session course. Irradiance is derived from the stated energy density per minute (0.09 J/cm^2^ ÷ 60 s); the corresponding illuminated area is approximately 5 cm^2^ per beam.

### 2.8. Outcome Measures

The primary efficacy endpoint was the proportion of participants achieving at least 30% reduction in VAS foot pain score from baseline to Week 3. Pain was characterized in three dimensions. Severity was quantified by the 0–100 mm VAS, on which a score of at least 50 mm was required for entry. Frequency was fixed by the eligibility criteria: all participants were required to have constant, daily foot pain of at least 3 months’ duration, so pain frequency was uniform across the cohort and did not differ between arms, and episodic or intermittent neuropathic pain was not studied. Pain type was characterized by the five domains of the NPSI, which distinguish superficial spontaneous burning pain, deep spontaneous pressing pain, paroxysmal (electric‐shock‐like or stabbing) pain, evoked (stimulus‐provoked) pain, and paresthesia/dysesthesia. Secondary outcomes included mean change in VAS pain score from baseline to Week 3 [17]; changes in neuropathic pain characteristics across the five domains of the Neuropathic Pain Symptom Inventory (NPSI: burning, pressing, paroxysmal, evoked, and paresthesia/dysesthesia [18]); patient satisfaction at Week 3 and Week 7 using a five‐point Likert scale; durability of treatment effects at Week 7, four weeks after treatment completion; blinding integrity; treatment adherence documented using a treatment administration log; and adverse events throughout the study.

### 2.9. Assessment of Harms

Potential risks were reviewed with each participant during the informed consent process and again at the pretreatment visit. Participants were instructed to record any event or observation that might constitute an adverse event, including new or worsening neuropathy symptoms, in a daily compliance log kept throughout the 3‐week treatment phase, and was given a 24‐h telephone number for the site. The investigator reviewed the completed log at the Week 3 visit and resolved ambiguous entries with the participant. In addition, the investigator performed a formal in‐person adverse event assessment at the Week 3 and Week 7 visits. Adverse events were recorded by the investigator, not by participant self‐assessment, on a case report form capturing expectedness, relatedness to the device or procedure, severity, seriousness, action taken, and outcome.

### 2.10. Statistical Analysis

The primary efficacy endpoint was analyzed in the intent‐to‐treat population using a chi‐square test comparing responder rates between groups. The observed between‐group responder‐rate difference was also assessed against the prespecified 35‐percentage‐point success criterion. ANCOVA with baseline VAS as a covariate was used to assess mean VAS change. Per‐protocol analyses were performed as sensitivity analyses. Secondary endpoints were summarized descriptively unless otherwise specified. Patient satisfaction was compared between groups using chi‐square tests at each time point. Spearman rank‐order correlation was used to examine the relationship between satisfaction and VAS change. A two‐sided alpha of 0.05 was used for inferential tests. The intent‐to‐treat population included all randomized participants, and the per‐protocol population included participants who completed all study procedures per protocol.

Missing data were handled as follows. Four participants, all randomized to sham treatment, withdrew after baseline assessment but before receiving the study device and had a valid baseline VAS but no Week 3 VAS value; no Week 3 values were missing in the active arm. Because withdrawal occurred before any device was issued, and therefore before any exposure to the intervention, these values were assumed to be missing completely at random. Each missing value was imputed as that participant’s baseline VAS minus the mean baseline‐to‐Week 3 VAS change observed among sham‐arm participants with complete data (−20.0 points; *n* = 27). Applying the responder criterion of at least a 30% reduction to the imputed values classified one of the four as a responder. A tipping point analysis spanning all four as nonresponders through all four as responders was also performed; the between‐group responder‐rate difference ranged from 43.7 to 30.8% points, and the prespecified 35‐percentage‐point criterion was met in three of the five scenarios. Per‐protocol analyses used observed data only. NPSI outcomes were prespecified as secondary and supportive and were analyzed on an available‐case basis restricted to participants with complete assessments at baseline, Week 3, and Week 7; they are reported descriptively without imputation and without adjustment for multiplicity, and no confirmatory inference is drawn from them.

## 3. Results

### 3.1. Participant Flow and Study Population

A total of 64 individuals were enrolled and randomized: 33 to active treatment and 31 to sham treatment, comprising the intent‐to‐treat population. Participant flow from screening through analysis is presented in Figure 1. Sixty‐seven individuals were assessed for eligibility and three were excluded for failure to meet inclusion criteria, two of whom had a screening VAS below the required 50 on the 0–100 scale. Enrollment was distributed across the four participating sites as follows: Miramar, Florida, *n* = 24; Dunedin, Florida, *n* = 15; Scottsdale, Arizona, *n* = 14; and West Covina, California, *n* = 11. Randomization was not stratified by site. Four participants in the sham arm did not complete study procedures per protocol, yielding a per‐protocol population of 60 participants (33 active and 27 sham). All four withdrew before receiving the study device, citing scheduled surgery, external time constraints, or the time commitment required by the study. No active‐treatment participants were excluded from the per‐protocol analysis at Week 3. A further eight participants, five active and three sham, were lost to follow‐up after the Week 3 endpoint assessment. Accordingly, 52 participants (28 active and 24 sham) contributed complete data at Week 7, and Week 7 analyses of NPSI, satisfaction, and blinding are based on this available‐case population. No participant discontinued because of a device‐ or treatment‐related serious adverse event.

### 3.2. Baseline Characteristics

Baseline demographic and clinical characteristics were comparable between groups with one exception (Table 1). Mean age was 67.4 ± 8.4 years in the active group and 64.7 ± 9.1 years in the sham group (*p* = 0.227). Baseline VAS pain scores were nearly identical between groups (76.5 ± 10.5 vs. 76.6 ± 14.1 mm; *p* = 0.976), indicating severe pain in both arms at entry. Pain frequency was uniform by design: every participant was required to have constant, daily foot pain of at least 3 months’ duration, and the mean duration of painful foot symptoms was 93.3 ± 89.9 months in the active group and 79.5 ± 97.6 months in the sham group (*p* = 0.557). The qualitative pain profile at baseline was also similar between arms and was dominated by paresthesia/dysesthesia (NPSI domain score 16.46 active vs 16.79 sham), followed by paroxysmal pain (12.00 vs 13.33), evoked pain (10.04 vs 13.88), pressing pain (9.07 vs 11.83), and burning pain (6.75 vs 6.63); the total baseline NPSI score was 54.32 in the active group and 62.46 in the sham group (Table 3). Concomitant analgesic and neuropathic‐pain medication use at baseline was comparable between arms: 19 of 33 active‐arm participants (58%) and 19 of 31 sham‐arm participants (61%) were taking at least one such medication (*p* = 0.762). Gabapentin was the most frequently used agent and was used to a comparable extent in both arms (15 of 33, 45%, vs 14 of 31, 45%; *z* = 0.024, *p* = 0.981), and no other individual medication was reported by more than three participants in either arm (Table 1). Two participants reported a physician‐initiated medication change during the treatment phase, made independently of study participation: an insulin dose reduction and the addition of allopurinol. Two further participants, one in each arm, recorded meloxicam as a mid‐study addition, but on verification both had been established on meloxicam before the treatment phase began and maintained a stable dose throughout, so neither represented a change from the baseline regimen. No participant changed a neuropathic‐pain medication or initiated a nonstudy pain‐management therapy. The duration since the diagnosis of diabetes was longer in the active group (15.6 ± 12.4 vs. 10.2 ± 7.9 years; *p* = 0.043).

**TABLE 3:** NPSI domain and total scores by treatment group across study evaluation periods (available‐case population).

Domain/statistic	Active (*n* = 28)			Sham (*n* = 24)		
	Baseline	Week 3	Week 7	Baseline	Week 3	Week 7
Burning pain, mean	6.75	3.36	3.43	6.63	5.58	4.96
Burning pain, SD	3.01	3.38	3.26	3.31	3.39	3.42
Pressing pain, mean	9.07	5.54	5.75	11.83	8.79	9.17
Pressing pain, SD	6.91	6.28	6.73	6.82	6.40	5.85
Paroxysmal pain, mean	12.00	7.39	6.93	13.33	10.08	9.92
Paroxysmal pain, SD	5.85	6.18	6.11	5.71	6.92	6.54
Evoked pain, mean	10.04	9.71	8.50	13.88	12.88	11.67
Evoked pain, SD	6.04	9.76	9.93	10.33	8.80	9.22
Paresthesia/dysesthesia, mean	16.46	10.32	8.54	16.79	12.67	13.42
Paresthesia/dysesthesia, SD	2.91	6.13	6.86	3.82	5.96	5.80
Total NPSI score, mean	54.32	36.32	33.14	62.46	50.00	49.13
Total NPSI score, SD	15.86	26.43	27.88	22.54	26.82	24.59

### 3.3. Treatment Adherence

Treatment adherence was high in the per‐protocol cohort. Forty‐nine of 60 participants (81.7%) reported completing all required at‐home treatment sessions. Among the remaining 11 participants, 24 sessions were self‐reported as missed during the 3‐week treatment phase, corresponding to an overall missed‐session rate of 0.95% among participants included in the per‐protocol adherence summary (24 missed sessions of 2520 planned sessions).

### 3.4. Primary Efficacy Outcome

In the intent‐to‐treat population, 72.7% of active‐treated participants (24/33) achieved the predefined responder criterion of at least 30% reduction in VAS pain score at Week 3, compared with 32.3% of sham‐treated participants (10/31). The responder‐rate difference was 40.5% points and exceeded the prespecified 35‐percentage‐point success criterion. Chi‐square analysis confirmed a statistically significant between‐group difference (*p* = 0.0024).

ANCOVA adjusted for baseline VAS demonstrated a mean VAS reduction of −33.4 points in the active group compared with −20.0 points in the sham group (between‐group difference, 13.4 points; *F* = 5.2; *p* = 0.026). Baseline pain intensity was not a significant predictor of outcome (*p* = 0.135).

Findings were consistent in the per‐protocol population. At Week 3, 72.7% of active‐treated participants (24/33) and 33.3% of sham‐treated participants (9/27) achieved the responder threshold (between‐group difference, 39.4% points; *p* = 0.0038). ANCOVA in the per‐protocol population confirmed a greater mean VAS reduction in the active group than in the sham group (*F* = 4.5; *p* = 0.038).

### 3.5. Pain Trajectory

VAS pain scores decreased progressively in the active group across study time points (Figure 2). Trajectory data are for the 52 participants with complete assessments through Week 7 (28 active and 24 sham), so the baseline values shown differ slightly from the intent‐to‐treat baseline reported in Table 1. By Day 10, mean pain scores had decreased by 20.9 points, with further reductions through Week 3 and maintenance of improvement through Week 7. Sham‐treated participants showed a smaller initial reduction followed by partial rebound during follow‐up. The net between‐group difference from baseline to Week 7 was 20.9 VAS points in favor of active treatment.

**FIGURE 2:**
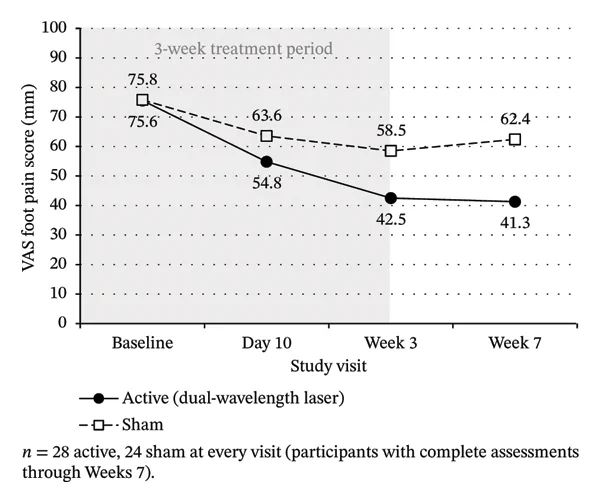
VAS foot pain trajectory across study time points by treatment group. Values are observed means for the 52 participants with complete assessments through Week 7 (28 active, 24 sham). VAS, visual analogue scale (0–100 mm).

### 3.6. NPSI

Among the 52 participants with complete NPSI assessments at baseline, Week 3, and Week 7 (28 active and 24 sham; see Participant flow and study population), active treatment was associated with consistent improvement across NPSI domains (Table 3; Figure 3). Burning pain decreased from 6.75 at baseline to 3.36 at Week 3 and 3.43 at Week 7 in the active group, compared with 6.63, 5.58, and 4.96 in the sham group. Paresthesia/dysesthesia scores also improved in the active group and remained improved at Week 7. Total NPSI score declined by 21.2 points in the active group compared with 13.3 points in the sham group through Week 7.

**FIGURE 3:**
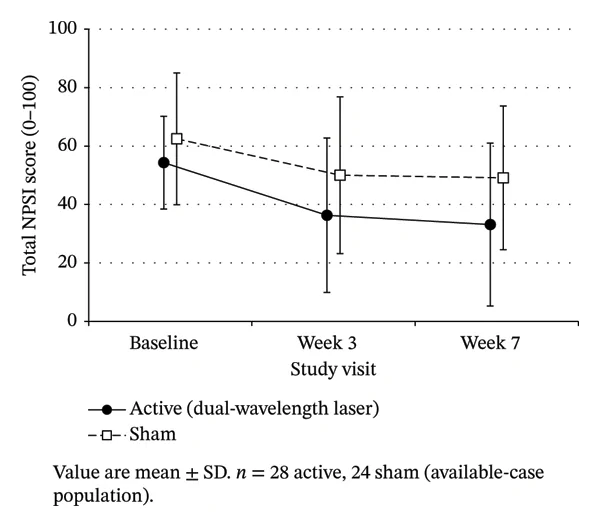
Total neuropathic pain symptom inventory scores by treatment group across study evaluation periods. Values are mean ± SD in the available‐case population (28 active, 24 sham). NPSI, neuropathic pain symptom inventory.

### 3.7. Patient Satisfaction

Satisfaction outcomes paralleled efficacy findings (Figure 4). Satisfaction was summarized on the available‐case population with complete assessments at Week 3 and Week 7 (28 active and 24 sham); the denominators therefore differ from those used for the primary VAS endpoint (33 and 31) and for blinding at Week 3 (33 and 27). At Week 3, 20 of 28 active‐treated participants (71.4%) reported being very satisfied or somewhat satisfied, compared with 10 of 24 sham‐treated participants (41.7%; *p* = 0.048). At Week 7, satisfaction was 21 of 28 (75.0%) in the active group compared with 8 of 24 (33.3%) in the sham group (*p* = 0.0047). Positive satisfaction ratings correlated with improvements in VAS pain scores at Week 3 (Spearman *r* = 0.70) and Week 7 (*r* = 0.61; both *p* < 0.0001).

**FIGURE 4:**
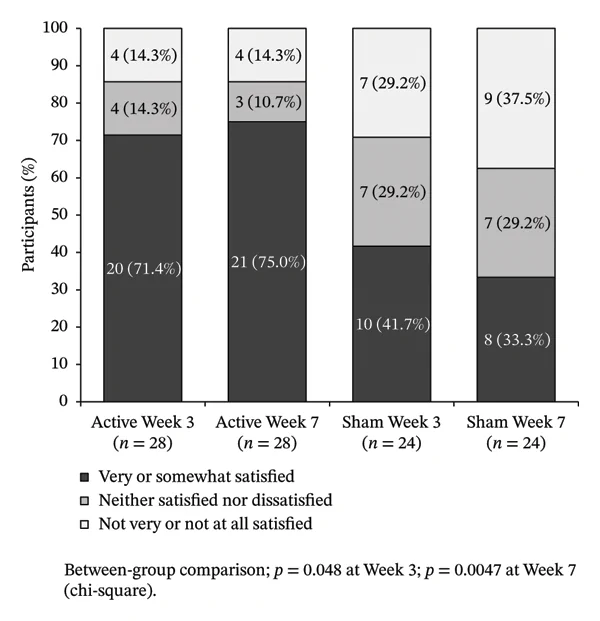
Proportion of participants reporting very satisfied or somewhat satisfied outcomes at Week 3 and Week 7. Bars show the number and percentage of participants in each satisfaction category in the available‐case population (28 active, 24 sham).

### 3.8. Blinding Validity

Perceived treatment allocation was recorded for 60 participants at Week 3 and 50 participants at Week 7; two participants with Week 7 outcome data did not complete the allocation‐perception item. At Week 3, 25 of 33 active‐arm participants (75.8%) and 14 of 27 sham‐arm participants (51.9%) correctly identified their allocation. At Week 7, 20 of 26 active‐arm participants (76.9%) and 16 of 24 sham‐arm participants (66.7%) did so. The association between actual and perceived allocation was statistically significant at both assessments (Week 3, *p* = 0.034; Week 7, *p* = 0.0039).

In the sham arm the Blinding Index was close to zero at Week 3 (+0.037; 95% CI −0.340 to +0.414), indicating guessing consistent with chance during the treatment phase, and rose to +0.333 (95% CI −0.044 to +0.711) at Week 7 as any placebo response receded. In the active arm the index exceeded zero at both assessments (Week 3, +0.515, 95% CI +0.223 to +0.808; Week 7, +0.538, 95% CI +0.215 to +0.862), indicating correct identification above chance. Participant explanations referred predominantly to perceived improvement or lack of improvement rather than to device or procedural cues, and no rationale indicated that the blind had been broken. Because allocation perception was recorded only after the treatment course had been completed, the index cannot distinguish inference drawn from a participant’s own symptom change from unmasking by another route.

### 3.9. Safety

No serious device‐ or treatment‐related adverse events were reported. Two nonserious adverse events, involving internal tingling and warmth during device use, were reported in 2 of 64 participants (3%) and were assessed as possibly device‐ and/or treatment‐related. No participant discontinued treatment because of a device‐ or treatment‐related adverse event.

## 4. Discussion

The main finding of this trial is that three weeks of twice‐daily, self‐administered dual‐wavelength LLLT relieved foot pain more than sham treatment in adults with painful DPN. At Week 3, 72.7% of active‐treated participants met the prespecified responder criterion of at least a 30% reduction in VAS pain, compared with 32.3% of sham‐treated participants, a difference of 40.5% points (*p* = 0.0024) that exceeded the prespecified 35‐percentage‐point success criterion, and mean VAS reduction was 33.4 points with active treatment versus 20.0 points with sham (*p* = 0.026). The effect persisted four weeks after treatment ended, with a net between‐group advantage of 20.9 VAS points at Week 7, and was accompanied by improvement across neuropathic symptom domains, higher treatment satisfaction, high adherence, and no serious device‐related adverse events. These results were consistent across all four participating sites.

These findings are relevant because painful DPN is often chronic, disabling, and incompletely controlled with pharmacological therapy. A nonpharmacological intervention that can be used at home under clinician direction may be particularly useful for patients with limited mobility, medication intolerance, or persistent symptoms despite standard care. The high adherence observed in this trial suggests that the treatment schedule was feasible for participants over the 3‐week treatment period.

The biological plausibility of LLLT in DPN is supported by the central role of mitochondrial dysfunction and oxidative stress in diabetic nerve injury. Peripheral nerve mitochondria are vulnerable to hyperglycemia‐associated impairment of oxidative phosphorylation, reduced ATP generation, and excessive reactive oxygen species production [3, 13, 19]. These processes can compromise axonal transport, ion‐channel homeostasis, myelin maintenance, microvascular support, and repair responses.

Red light at approximately 635–640 nm is commonly discussed in photobiomodulation as interacting with cytochrome c oxidase, the terminal oxidase of the mitochondrial electron transport chain [10, 12]. Absorption at this level may influence electron transfer, mitochondrial membrane potential, ATP production, and nitric‐oxide‐related vascular signaling. The 405 nm violet wavelength may provide a mechanistically distinct input through absorption by flavoproteins, porphyrins, and other redox‐active chromophores involved in upstream electron handling and oxidative signaling [20, 21].

The rationale for combining 640 red and 405 nm violet coherent wavelengths is that they may engage complementary photochemical targets within bioenergetic and redox pathways. In DPN, where impaired energy metabolism and oxidative stress contribute to distal nerve dysfunction, a multi‐target photobiomodulatory signal may reduce nociceptive sensitization and support tissue conditions favorable to peripheral nerve maintenance. These mechanistic interpretations remain hypothesis‐generating, however, because this clinical trial did not include direct mitochondrial, electrophysiological, or structural nerve endpoints.

The dorsal surface of the foot was selected because it is accessible for noncontact self‐administration at home and because it overlies the neural structures implicated in painful DPN. The laser lines extended from the anterior ankle across the mid‐dorsum to the toes. Along nearly this entire course, the medial and intermediate dorsal cutaneous nerves, the terminal branches of the superficial fibular (peroneal) nerve, lie subcutaneously immediately beneath the treated skin. At the anterior ankle the parent trunk is at its most superficial, so the field also included nerve tissue proximal to the territory in which participants reported symptoms [16]. A clinically instructive precedent for treating adjacent to, rather than at, the symptomatic surface comes from a randomized, placebo‐controlled study of 635 nm red laser therapy for temporary relief of pain associated with plantar fasciitis. In that trial, irradiation was applied to the tarsal tunnel region rather than to the painful plantar heel directly, yet participants experienced significant reductions in plantar heel pain [22]. This supports the view that photobiomodulatory effects need not be confined to the irradiated surface, and it informed the decision to treat the dorsum in the present trial.

The NPSI data suggest that the benefit was not uniform across symptom types. Between baseline and Week 7, active treatment produced larger reductions than sham in paresthesia/dysesthesia (7.92 vs 3.37 points, or 48% vs 20% of the baseline domain score), burning pain (3.32 vs 1.67 points; 49% vs 25%), paroxysmal pain (5.07 vs 3.41 points; 42% vs 26%), and pressing pain (3.32 vs 2.66 points; 37% vs 22%). Evoked pain was the exception: reductions were small and closely similar in the two arms (1.54 vs 2.21 points; 15% vs 16%), so no differential effect on stimulus‐provoked pain was apparent. The benefit therefore appears concentrated on spontaneous ongoing pain, paroxysmal pain, and abnormal nonpainful sensations rather than on stimulus‐evoked pain, which is consistent with an effect on ectopic spontaneous discharge in the irradiated nerve segment without normalization of peripheral or central sensitization. Because these domain‐level comparisons were available—case secondary analyses that were neither powered as confirmatory endpoints nor adjusted for multiplicity—they are hypothesis‐generating; a trial powered on NPSI domains and incorporating quantitative sensory testing would be needed to establish symptom‐specific efficacy. Nevertheless, the consistency of the VAS, NPSI, satisfaction, and durability findings supports the clinical relevance of the observed treatment effect.

Compared with first‐line pharmacological therapies, the safety profile observed in this trial is notable. No serious device‐related adverse events occurred, and only two nonserious events were reported as possibly related to the device or treatment. This favorable tolerability profile may be clinically important for patients who experience adverse effects from medications or seek adjunctive nondrug options.

The study also supports the feasibility of a prescription home‐use model for painful DPN. Participants completed treatment independently after standardized instruction, and adherence was high. The results therefore suggest that home photobiomodulation can be integrated into physician‐directed management without requiring frequent in‐office treatments during the active treatment period.

Dual‐wavelength laser therapy is also a simple treatment option to add to daily practice. The clinician prescribes the course; the patient then performs all 42 treatments at home, twice daily across three weeks, so no in‐office treatment time is required. No training session was provided: participants operated the device from the written proper use reference guide alone, and the adherence and treatment effect observed here suggest that these instructions were sufficient for unsupervised home use. The device labeling was subsequently reviewed by the FDA in the course of clearance for prescription home use (K251903). The device has fixed output with no dose to select, uses no consumables, and is held away from the skin, so there is nothing for the clinician to titrate. Adherence was high without additional support: 49 of 60 participants completed every scheduled session and fewer than 1% of scheduled sessions were missed. Review can therefore take place at ordinary follow‐up intervals, and the clinical decisions are limited to patient selection and to confirming adherence and response.

### 4.1. Limitations

The primary limitation is the duration of follow‐up. Outcomes were assessed through Week 7, capturing durability four weeks after completion of the 3‐week treatment course. Painful DPN is a chronic condition, and longer follow‐up is needed to determine the persistence of benefit, the optimal retreatment interval, and the effects of repeated treatment courses. Because assessment ended at Week 7, these results support a conclusion of short‐term pain relief only.

Replication in other settings is subject to practical constraints. The device is a fixed‐output dual‐wavelength unit supplied by the sponsor and is not interchangeable with other photobiomodulation devices, so these results should not be assumed to transfer to devices with different wavelengths, coherence, irradiance, or beam geometry; because output is fixed and no dose adjustment is possible, replication depends on access to the same device rather than on reproducing a dose from published parameters. The regimen requires twice‐daily self‐treatment for 21 consecutive days, which places a sustained adherence burden on participants and depends on the validated written instructions and on patients being able to position the device unaided; individuals with significant hand‐dexterity limitations were excluded, and the findings may not extend to them. All four sites were U.S. outpatient specialty practices, so performance in primary care, in inpatient settings, or in health systems with different access to prescription home‐use devices remains untested. Within those constraints the protocol itself is reproducible: all parameters required for replication are reported in Table 2, and the eligibility criteria, randomization and concealment procedures, sham design, endpoint definitions, missing‐data rules, and analysis plan are reported in full here and were registered before enrollment.

Finally, concomitant analgesic regimens were required to remain stable and were monitored throughout, but they were neither randomized nor adjusted for analytically, so these results describe the intervention as an addition to existing therapy rather than its effect in isolation, and no conclusion can be drawn about interaction with specific drug classes.

## 5. Conclusion

In adults with painful DPN, three weeks of twice‐daily home use of a dual‐wavelength 640 nm red and 405 nm violet low‐level laser reduced foot pain more than sham treatment, and the benefit persisted four weeks after treatment ended with no serious device‐related adverse events. Dual‐wavelength LLLT is therefore a well‐tolerated complementary option for the temporary relief of painful DPN alongside standard care; longer follow‐up and objective peripheral nerve endpoints are needed to establish durability.

## Author Contributions

Sandra Franco, Jaime Franco, Cesar Lara, Kirk Gair, and Kerry Zang contributed to investigation and clinical conduct. Steve Shanks and Travis Sammons contributed to study administration and manuscript preparation. All authors contributed to interpretation of findings, reviewed the manuscript, and agree to be accountable for the work.

## Funding

This clinical study was sponsored by Erchonia Corporation, Fountain Inn, SC, USA, which provided the study devices, supported site training and study monitoring, and covered study‐related costs. No external grant funding was received.

## Disclosure

The clinical data from this trial supported U.S. FDA 510(k) clearance K251903 for the Erchonia DPN Laser (Model EVRL) for prescription home use as an adjunctive treatment for temporary relief of diabetic peripheral neuropathy foot pain [23]. All authors approved the final version.

## Ethics Statement

The study protocol was approved by an independent institutional review board (WCG Copernicus Group; IRB Tracking #20220447). All participants provided written informed consent before enrollment.

## Conflicts of Interest

Travis Sammons and Steve Shanks are employees of the study sponsor, Erchonia Corporation. Erchonia Corporation supplied study devices, supported site training, and participated in manuscript preparation. Sandra Franco, Jaime Franco, Cesar Lara, Kirk Gair, and Kerry Zang served as independent investigators at their respective sites and reported no additional conflicts of interest related to this manuscript. The statistical analysis for the study was performed by an independent statistical consultant, Elvira Cawthon.

## Supporting Information

Additional supporting information can be found online in the Supporting Information section.

## Supporting information


**Supporting Information** A completed CONSORT 2025 checklist (Supporting File 1: CONSORT 2025 Checklist.docx), indicating the page on which each checklist item is addressed in this manuscript, is provided as supporting information accompanying this article [15].

## Data Availability

Data are available on request from the authors.
