# Supplementary material for: Dual‐Wavelength Laser Therapy for Painful Diabetic Peripheral Neuropathy: A Randomized, Double‐Blind, and Sham‐Controlled Trial
Source: Pain Res Manag. 2026 Oct 11;2026:8710857. doi: 10.1155/prm/8710857 (PMC13655509; doi:10.1155/prm/8710857)
Supplement: Supplementary file 1 [file PRM-2026-8710857-s001.docx]

**CONSORT 2025 checklist of information to include when reporting a randomised trial**

Manuscript title: Dual-Wavelength Laser Therapy for Painful Diabetic Peripheral Neuropathy: A Randomized, Double-Blind, Sham-Controlled Trial

Manuscript ID: 8710857

Corresponding author: Travis Sammons, Erchonia Corporation, Fountain Inn, SC, USA

Page numbers refer to the revised manuscript submitted with this checklist.

| **Section/topic** | **No** | **CONSORT 2025 checklist item description** | **Reported on page No** |
| --- | --- | --- | --- |
| **Title and abstract** | | | |
| Title and structured abstract | 1a | Identification as a randomised trial | Page 1 |
|  | 1b | Structured summary of the trial design, methods, results, and conclusions | Pages 1-2 |
| **Open science** | | | |
| Trial registration | 2 | Name of trial registry, identifying number (with URL) and date of registration | Pages 1, 3, 17 (ClinicalTrials.gov NCT05291975; registered before enrollment of the first participant) |
| Protocol and statistical analysis plan | 3 | Where the trial protocol and statistical analysis plan can be accessed | Pages 3, 17 (protocol, statistical analysis plan, and informed consent form are attached to the ClinicalTrials.gov registry record, NCT05291975) |
| Data sharing | 4 | Where and how the individual de-identified participant data (including data dictionary), statistical code and any other materials can be accessed | Page 17 (data availability statement: "Data available on request from the authors.") |
| Funding and conflicts of interest | 5a | Sources of funding and other support (e.g., supply of drugs), and role of funders in the design, conduct, analysis and reporting of the trial | Page 17 |
|  | 5b | Financial and other conflicts of interest of the manuscript authors | Page 16 |
| **Introduction** | | | |
| Background and rationale | 6 | Scientific background and rationale | Pages 2-3 |
| Objectives | 7 | Specific objectives related to benefits and harms | Page 3 |
| **Methods** | | | |
| Patient and public involvement | 8 | Details of patient or public involvement in the design, conduct and reporting of the trial | Page 4 |
| Trial design | 9 | Description of trial design including type of trial (e.g., parallel group, crossover), allocation ratio, and framework (e.g., superiority, equivalence, non-inferiority, exploratory) | Page 3 |
| Changes to trial protocol | 10 | Important changes to the trial after it commenced including any outcomes or analyses that were not prespecified, with reason | Page 3 (no changes to eligibility criteria, outcomes, or design; no interim analyses) |
| Trial setting | 11 | Settings (e.g., community, hospital) and locations (e.g., countries, sites) where the trial was conducted | Page 3 (four U.S. outpatient sites in Florida, California, and Arizona) |
| Eligibility criteria | 12a | Eligibility criteria for participants | Pages 3-4 |
|  | 12b | If applicable, eligibility criteria for sites and for individuals delivering the interventions (e.g., surgeons, physiotherapists) | Page 4 |
| Intervention and comparator | 13 | Intervention and comparator with sufficient details to allow replication. If relevant, where additional materials describing the intervention and comparator (e.g., intervention manual) can be accessed | Pages 4-5; Table 1, page 5 |
| Outcomes | 14 | Prespecified primary and secondary outcomes, including the specific measurement variable, analysis metric, method of aggregation, and time point for each outcome | Pages 5-6 |
| Harms | 15 | How harms were defined and assessed (e.g., systematically, non-systematically) | Page 6 |
| Sample size | 16a | How sample size was determined, including all assumptions supporting the sample size calculation | Page 3 |
|  | 16b | Explanation of any interim analyses and stopping guidelines | Page 3 (no interim analyses; no stopping guidelines) |
| Randomisation: sequence generation | 17a | Who generated the random allocation sequence and the method used | Page 4 |
|  | 17b | Type of randomisation and details of any restriction (e.g., stratification, blocking and block size) | Page 4 |
| Allocation concealment mechanism | 18 | Mechanism used to implement the random allocation sequence (e.g., central computer/telephone; sequentially numbered, opaque, sealed containers), describing any steps to conceal the sequence until interventions were assigned | Page 4 |
| Implementation | 19 | Whether the personnel who enrolled and those who assigned participants to the interventions had access to the random allocation sequence | Page 4 |
| Blinding | 20a | Who was blinded after assignment to interventions (e.g., participants, care providers, outcome assessors, data analysts) | Page 4 |
|  | 20b | If blinded, how blinding was achieved and description of the similarity of interventions | Page 4 |
| Statistical methods | 21a | Statistical methods used to compare groups for primary and secondary outcomes, including harms | Pages 6-7 |
|  | 21b | Definition of who is included in each analysis (e.g., all randomised participants), and in which group | Pages 6-7 |
|  | 21c | How missing data were handled in the analysis | Page 7 |
|  | 21d | Methods for any additional analyses (e.g., subgroup and sensitivity analyses), distinguishing prespecified from post hoc | Pages 6-7 |
| **Results** | | | |
| Participant flow, including flow diagram | 22a | For each group, the numbers of participants who were randomly assigned, received intended intervention, and were analysed for the primary outcome | Pages 7-8; Figure 1, page 8 |
|  | 22b | For each group, losses and exclusions after randomisation, together with reasons | Pages 7-8; Figure 1, page 8 |
| Recruitment | 23a | Dates defining the periods of recruitment and follow-up for outcomes of benefits and harms | Page 3 (first enrolled 14 June 2022; last enrolled 25 September 2024; final Week 7 assessment 14 November 2024) |
|  | 23b | If relevant, why the trial ended or was stopped | Page 3 (planned enrollment target reached) |
| Intervention and comparator delivery | 24a | Intervention and comparator as they were actually administered (e.g., where appropriate, who delivered the intervention/comparator, how participants adhered, whether they were delivered as intended (fidelity)) | Pages 4-5 (self-administered at home), 9 (adherence) |
|  | 24b | Concomitant care received during the trial for each group | Page 4 (stable analgesic regimen required; no non-study pain therapies); Table 2, pages 9-10 |
| Baseline data | 25 | A table showing baseline demographic and clinical characteristics for each group | Table 2, pages 9-10 |
| Numbers analysed, outcomes and estimation | 26 | For each primary and secondary outcome, by group: the number of participants included in the analysis; the number with available data at the outcome time point; result for each group and the estimated effect size and its precision (such as 95% confidence interval); for binary outcomes, presentation of both absolute and relative effect size | Pages 10-13; Table 3, page 11; Figures 2-4, pages 10, 12, 13 |
| Harms | 27 | All harms or unintended events in each group | Page 14 |
| Ancillary analyses | 28 | Any other analyses performed, including subgroup and sensitivity analyses, distinguishing pre-specified from post hoc | Pages 10-13 (per-protocol and ANCOVA analyses, identified as prespecified supportive analyses) |
| **Discussion** | | | |
| Interpretation | 29 | Interpretation consistent with results, balancing benefits and harms, and considering other relevant evidence | Pages 14-16 |
| Limitations | 30 | Trial limitations, addressing sources of potential bias, imprecision, generalisability, and, if relevant, multiplicity of analyses | Page 16 |
